# Bilateral Ulcerative Carpal Tunnel Syndrome: A Case Report

**DOI:** 10.7759/cureus.117547

**Published:** 2026-10-06

**Authors:** Sydney J Schwab, Elena Gonzalez Caldito

**Affiliations:** 1 Dermatology, Louisiana State University Health Sciences Center Shreveport, Shreveport, USA

**Keywords:** diabetes mellitus, digital ulceration, median nerve, thenar atrophy, ulcerative carpal tunnel syndrome

## Abstract

Ulcerative carpal tunnel syndrome (UCTS) is a rare, cutaneous manifestation of severe median neuropathy. We present a woman in her 60s with poorly controlled type 2 diabetes mellitus with an HbA1c of 9.5 who reported painless vesicles and presented with erosions involving bilateral median nerve distribution. Examination showed erosions with re-epithelialization of the phalanges and bilateral thenar atrophy, with associated nocturnal paresthesias. Nerve conduction studies confirmed severe bilateral median neuropathy at the wrists, consistent with carpal tunnel syndrome. Surgical decompression was recommended but deferred pending improved glycemic control, and conservative management was pursued. This case highlights the importance of recognizing UCTS in patients with diabetes and digital ulcerations. Careful assessment of lesion distribution may help distinguish UCTS from more common diabetic neuropathic or microvascular complications and prevent delayed diagnosis.

## Introduction

Ulcerative carpal tunnel syndrome (UCTS) is a rare and advanced manifestation of carpal tunnel syndrome (CTS), the most common entrapment neuropathy defined by median nerve compression. Sensory impairment in the median nerve distribution can result in hypoesthesia and loss of protective sensation, predisposing the affected digits to repetitive trauma and impaired wound healing [1]. Autonomic dysfunction and impaired microvascular regulation have also been proposed as mechanisms that contribute to ulceration and impaired wound healing, although they are less well established in UCTS [1]. As a result, patients with UCTS present with painless ulcerations typically involving the palmar aspect of the distal first through third digits in the median nerve distribution accompanied by atrophy of the thenar eminence.

We report a case of bilateral UCTS in a woman with poorly controlled type 2 diabetes mellitus who reported recurrent vesicles and presented with erosions in the distribution of the median nerve with severe atrophy of the thenar eminences. Nerve conduction studies (NCS) confirmed suspected bilateral carpal tunnel syndrome. This patient’s reported history of vesicles preceding erosions and ulcerations bilaterally represents an unusual presentation of ulcerative carpal tunnel syndrome.

## Case presentation

A woman in her 60s with poorly controlled type 2 diabetes mellitus with an HbA1c of 9.5 presented with a 1-year history of reported painless, intermittent, clear fluid-filled vesicles that would come and go, leading to erosions localized to the phalanges of the first through third digits of the left hand and the second through third digits of the right hand. The lesions were primarily located on the palmar side of the mentioned left distal digits and the subungual region of the mentioned right distal digits. A review of systems was notable for nocturnal paresthesias in the median nerve distribution bilaterally, causing sleep disruption that improved only with repositioning of her hands. Sensory loss was present, and Phalen's test was positive. Examination of the hands revealed erosions with re-epithelialization on the phalanges and moderate-to-severe atrophy of the thenar eminences (Figure 1). UCTS vs neuropathic etiology was on the differential. Given the involvement of the palmar aspect of the digits, a high-potency topical corticosteroid, clobetasol ointment, was prescribed in an attempt to relieve symptoms. Silver sulfadiazine was prescribed as wound care to minimize infection in the interim. This provided some relief, but symptoms were still present when the patient presented at her one-month follow-up. Examination showed improved erosions with persistent thenar eminence atrophy of the left hand (Figure 2). Ulceration of the distal third digit of the right hand was present (Figure 3). In addition, the patient's lesions episodically showed clinical concern for a superimposed bacterial infection requiring multiple courses of sulfamethoxazole-trimethoprim.

**Figure 1:**
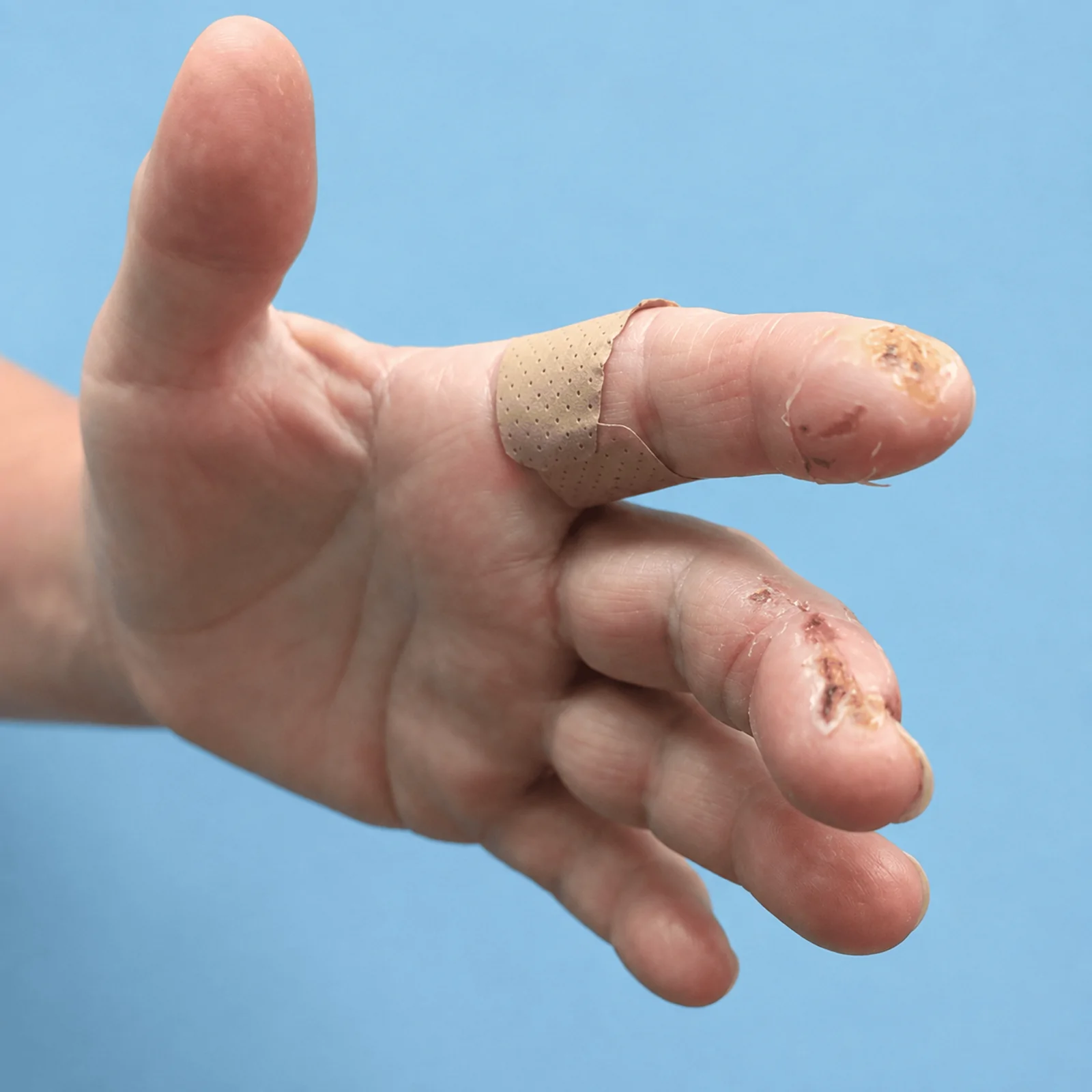
Erosions with re-epithelialization of the phalanges of the left hand before treatment with silver sulfadiazine cream, clobetasol ointment, and conservative management

**Figure 2:**
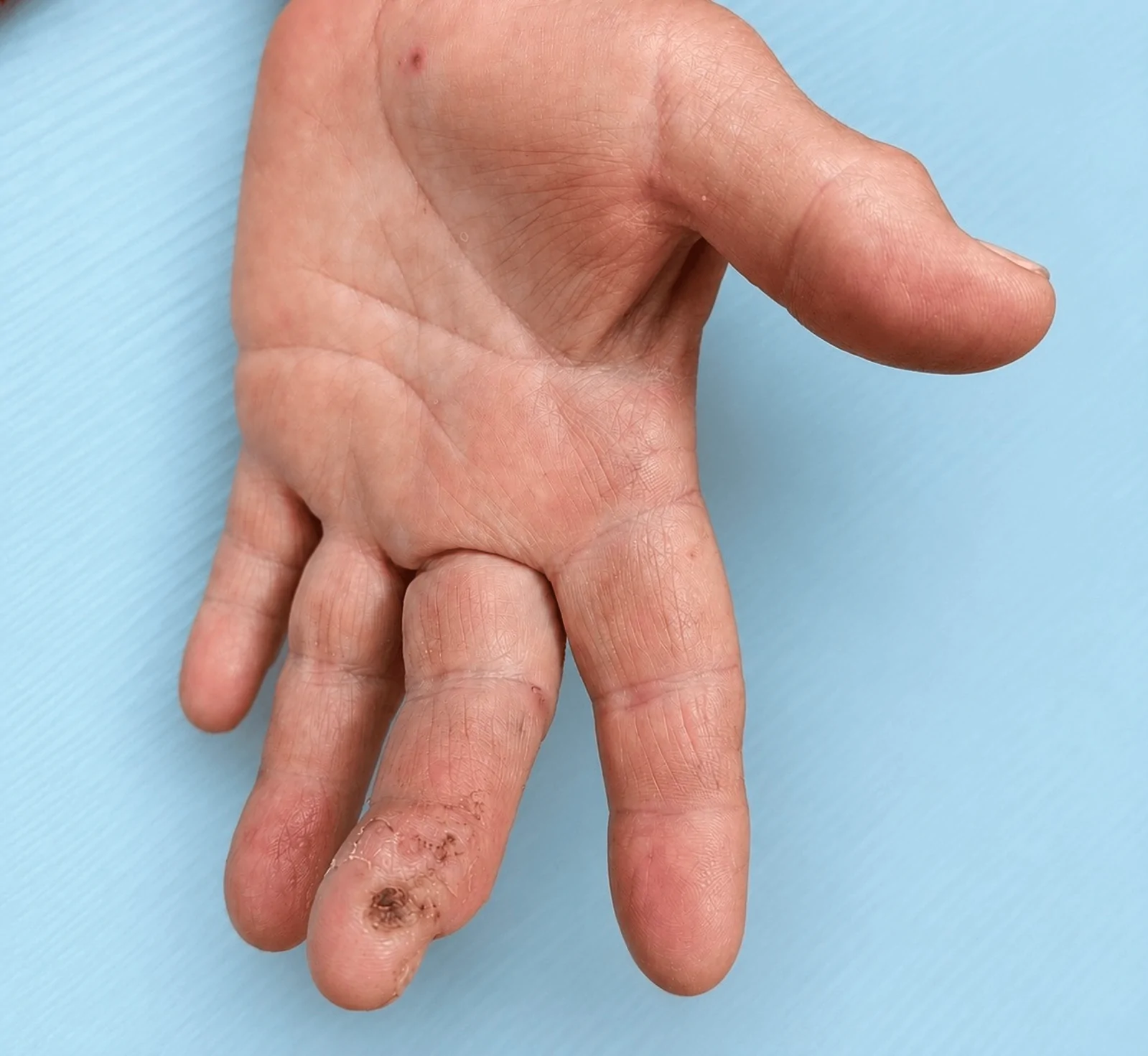
Improved erosions and ulcerations of the left hand following conservative treatment, with persistent thenar eminence atrophy

**Figure 3:**
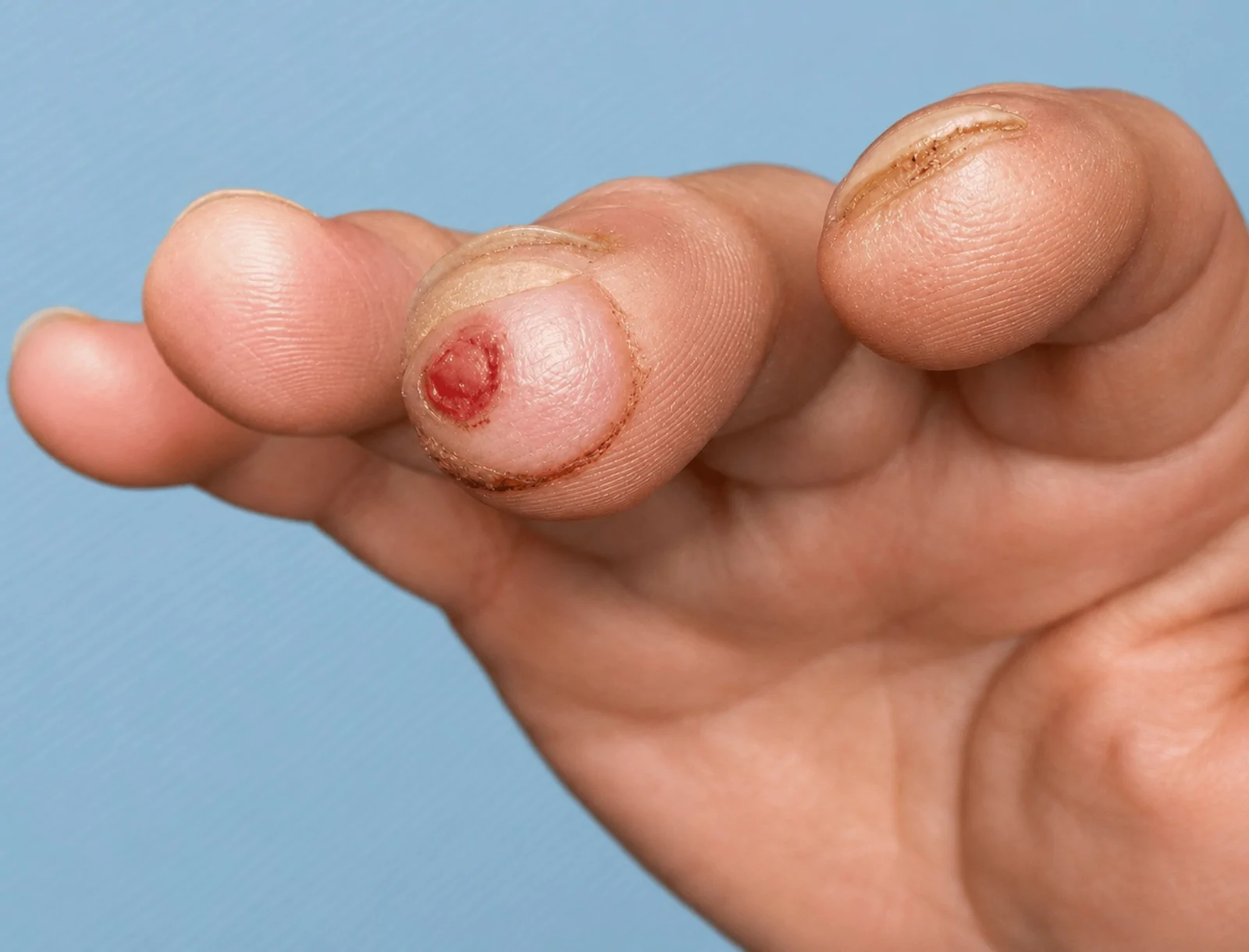
Ulceration of the distal third digit of the right hand

Given the characteristic acral distribution of the vesicles along the median nerve combined with severe symptoms of carpal tunnel syndrome, a diagnosis of UCTS was then pursued. Electrodiagnostic studies, including nerve conduction studies (NCS), were obtained and demonstrated severe bilateral demyelinating median neuropathy at the wrists, interpreted by the neurologist. The amplitude and conduction velocity of the left median motor nerve were decreased (Table 1). The left median sensory nerve showed no response, and the right median sensory nerve showed prolonged distal peak latency, reduced amplitude, and decreased conduction velocity (Table 2). The motor and sensory components of the ulnar nerve and the sensory component of the radial nerve were within normal limits on her NCS, which supports sparing of those digits symptomatically. NCS findings confirmed bilateral carpal tunnel syndrome, more severe on the left hand, which was consistent with her cutaneous manifestations. Following evaluation by a hand surgeon, surgical intervention was discussed and stated as the preferential treatment. The risks and benefits of carpal tunnel release were discussed between the patient and hand surgeon and deferred until improved glycemic control could be achieved due to the higher risk of infection and wound complications. Conservative treatment was recommended in the meantime with wrist splinting and a carpal tunnel injection. The patient tolerated the corticosteroid injections well, and her blood sugar remained stable. This treatment has served as a bridge while she continues to optimize her HbA1c for eventual surgical decompression.

**Table 1:** Abnormal nerve conduction study results of the left median motor nerve F-Lat: F-wave latency; CV: conduction velocity; ms: millisecond; Norm: normal; mV: millivolts; cm: centimeter; APB: abductor pollicis brevis

Site	Latency		Amplitude		Total Duration	F-Lat	Segment	Distance	CV	
	(ms)	Norm	(mV)	Norm	(ms)	(ms)	-	(cm)	(ms)	Norm
Left Median (APB) Motor										
Wrist	3.3	<4.4	1.6, 2	>4.0	20	35.1	-	-	-	-
Elbow	7.7	-	1.5, 6	-	21	-	Elbow-Wrist	20	45	>49

**Table 2:** Abnormal nerve conduction study results of the bilateral median sensory nerve μV: microvolt; m/s: meter/second; NR: no response

Site	Negative Peak Latency		Amplitude (O-P)		Total Duration (ms)	Segment	Distance (cm)	Velocity (m/s)
	(ms)	Norm	(μV)	Norm				
Left Median Sensory								
Wrist-Digit II	NR	<3.5	NR	>20	NR	Wrist-Digit II	13	NR
Right Median Sensory								
Wrist-Digit II	5.1	<3.5	14	>20	4.1	Wrist-Digit II	13	25

## Discussion

UCTS is a rare manifestation of longstanding median nerve compression causing autonomic and sensory dysfunction in the affected digits [2]. Sensory loss and sympathetic dysregulation can lead to repetitive trauma with subsequent ulceration [3]. If left untreated, patients can develop complications, including secondary bacterial infections of the ulcerations and nail involvement, for example, dystrophy and onycholysis. Progression to a mutilating form can develop if left untreated, involving acro-osteolysis, which can subsequently result in amputation [2,4,5]. UCTS is a clinical diagnosis supported by NCS findings demonstrating prolonged median sensory and motor latencies at the wrist. Digital ulcers have a broad differential diagnosis including scleroderma, herpetic whitlow, vasculitis, diabetic neuropathy, Raynaud phenomenon, bullosis diabeticorum, and trauma [1]. Autoimmune blistering disorders and vasculitic ulcers should be excluded if any clinical suspicion is present [4]. UCTS can be mistaken for other causes of neuropathic or ischemic acral ulcerations, so attention to distribution is necessary for accurate diagnosis [4].

Distinguishing UCTS from ulcerations related to diabetic neuropathy in patients with uncontrolled diabetes mellitus can be clinically challenging. Although diabetes mellitus is a well-recognized risk factor for CTS, its association with UCTS remains unknown [6]. The rarity of UCTS limits available data to make associations with potential risk factors and diseases. Uncontrolled diabetes may lead to digital ulceration as a complication of neuropathy or microvascular disease, but the distribution and associated median neuropathy should prompt consideration of UCTS. Incorrectly identifying the cutaneous lesions as a manifestation of uncontrolled diabetes can delay recognition of the correct pathology. Careful evaluation of lesion distribution is essential, and the correct correlation of clinical and electrophysiologic studies, especially in patients with uncontrolled diabetes, is necessary to avoid overlooking UCTS. Our case highlights the importance of recognizing the median nerve distribution rather than attributing the patient's erosions to diabetes-associated cutaneous complications. 

UCTS responds best to surgical release of the median nerve entrapment, and skin manifestations typically resolve [2,3]. Gomes et al. described a case series where patients with UCTS demonstrated classic paresthesias and erosions of the first three phalanges and NCS findings consistent with median nerve neuropathy. Most patients underwent surgical treatment and reported complete resolution of cutaneous manifestations [3]. The rarity of UCTS can lead to diagnostic delay, highlighting the importance of physician education and exposure to atypical disease. This case highlights the rare cutaneous manifestations and complications of advanced carpal tunnel syndrome. Gomes et al. describe the largest published case series of UCTS, where most cases are unilateral [3]. This case represents an atypical bilateral, vesicular presentation which highlights diagnostic complexity. Limitations of this case report include lack of photo documentation of the vesicular lesions reported by the patient. The patient has not yet undergone surgical decompression; therefore, the long-term response to definitive treatment and potential resolution or recurrence of the ulcerations cannot yet be assessed. This case and its limitations represent a realistic challenge in the management of advanced UCTS, balancing the urgency of surgical decompression with the need for preoperative glycemic optimization. Conservative treatment may provide limited benefit in advanced UCTS; however, risks and benefits of carpal tunnel release must be weighed through shared decision-making between the patient and the physician.

## Conclusions

UCTS is a cutaneous manifestation of carpal tunnel syndrome that may be overlooked because of its atypical presentation. Clinicians should include UCTS in their differential diagnoses when patients present with recurrent, painless erosions and ulcerations in the median nerve distribution. Digital ulceration can get overlooked in patients with uncontrolled diabetes, as this could be a complication of their neuropathy and microvascular disease. Sensory abnormalities and thenar eminence atrophy may be present, supporting the diagnosis. Early recognition is essential for the prevention of tissue injury and the development of the mutilating form, which can lead to auto-amputation of the affected digits. 

## References

[1] Iyer VG (2023). Clinical reasoning: a septuagenarian with painless ulceration on the fingertip. Neurology.

[2] Natale M, Spennato P, Bocchetti A, Fratta M, Savarese L, Rotondo M (2005). Ulcerative and mutilating variant of carpal tunnel syndrome. Acta Neurochir (Wien).

[3] Gomes TF, Kieselova K, Teixeira V, Pereira N, Santiago F, Henrique M (2021). Clinical characteristics of ulcerating carpal tunnel syndrome among older adults in Portugal. JAMA Dermatol.

[4] Carmen MB, Ernesto G (2022). Ulcerating variety of carpal tunnel syndrome mimicking scleroderma. MOJ Orthop Rheumatol.

[5] Escolà H (2024). Acral ulcerations: an unusual presentation of carpal tunnel syndrome. J Clin Images Med Case Rep.

[6] Padua L, Cuccagna C, Giovannini S (2023). Carpal tunnel syndrome: updated evidence and new questions. Lancet Neurol.

